# PET Waste-Derived Hard Carbon with Superior Rate Capability for Sodium-Ion Battery Anodes

**DOI:** 10.3390/ma19122457

**Published:** 2026-06-08

**Authors:** Aizhuz Sarsengaliyeva, Aliya Mukanova, Sung-Soo Kim, Arailym Nurpeissova

**Affiliations:** 1 Institute of Batteries (IoB), Kabanbay Batyr Ave. 53, Astana 010000, Kazakhstan; saizhuzzaa@gmail.com (A.S.); aliya.mukanova@nu.edu.kz (A.M.); 2 Institute of New Materials and Energy Technologies, Kabanbay Batyr Ave. 53, Astana 010000, Kazakhstan; 3 Graduate School of Energy Science and Technology, Chungnam National University, 99 Daehak-ro, Yuseong-gu, Daejeon 34134, Republic of Korea

**Keywords:** polyethylene terephthalate (PET), plastic waste, hard carbon (HC), sodium-ion battery (SIB) anode material

## Abstract

Sodium-ion batteries (SIBs) are considered a promising alternative to lithium-ion systems, with hard carbon (HC) being the most suitable anode material due to its disordered structure and increased interlayer distance. At the same time, the recycling of polyethylene terephthalate (PET), whose waste volumes are constantly growing, remains a pressing issue. In this work, recycled PET is used as a precursor for obtaining HC by direct carbonization at temperatures of 900–1400 °C. It is shown that the carbonization temperature significantly affects the structure and electrochemical properties of the obtained materials. The best characteristics were demonstrated by samples PET_1000 and PET_1100, which provided a high reversible capacity of 275–280 mAh g^−1^ at a current density of 20 mA g^−1^ in sodium-ion half-cells. The results confirm that controlled carbonization of recycled PET is an effective approach for obtaining highly efficient anode materials for SIBs and, at the same time, represents a promising way to utilize plastic waste.

## 1. Introduction

The development of efficient and environmentally friendly energy storage systems has become increasingly critical in response to rising global energy consumption and the urgent demand for sustainable development [1]. Lithium-ion batteries (LIBs) currently dominate the energy storage market [2]; however, their large-scale deployment is constrained by the limited and uneven distribution of lithium resources, as well as the environmental impact associated with the extraction and end-of-life disposal of lithium [3]. Owing to the natural abundance and low cost of sodium, sodium-ion batteries (SIBs) have emerged as a promising alternative and have attracted considerable research attention in recent years [4]. SIBs also use a “rocking chair” architecture similar to LIBs, which allows the lithium industry’s achievements to be quickly transferred to sodium technologies [5]. In addition, SIBs typically demonstrate higher safety and better performance at low temperatures, which is important for stationary energy storage systems [6].

While searching for alternative energy storage systems, the world is facing an acute problem of plastic waste accumulation, especially PET [7]. PET, also referred to by its recycling resin identification code #1, is one of the most widely used thermoplastic polymers; however, most of it is not disposed of properly, posing a serious threat to the environment [8]. It belongs to the polyester family, which is characterized by the presence of ester linkages in the main polymer backbone [9]. According to production statistics categorized by industrial application sectors, drinking water bottles and containers account for approximately 26% of total PET consumption, followed by carbonated soft drink bottles (26%), other beverages and juices (18%), sheets and films (14%), food packaging (9%), and non-food applications, such as cosmetics (6%) [10,11].

Hard carbon (HC) is recognized as one of the most promising anode materials for sodium-ion batteries [12]. Its predominantly amorphous structure, with graphite-like areas and micropores, enables efficient sodium storage through a combination of intercalation and pore-filling mechanisms involving sodium clustering [13]. Unlike the graphite anode used in LIBs, HC has a more disordered structure with enlarged interlayer spacing (~0.36–0.42 nm), which allows it to provide high capacity and low-voltage plateau discharge [14]. Furthermore, the presence of numerous defects provides additional active sites and fast Na^+^ transport pathways [15]. Carbon-based anodes also exhibit good cycling stability, low sodium storage potential, and low material cost, making them highly attractive for sodium-ion battery applications [16].

Mechanical and chemical recycling methods often have drawbacks, such as deterioration of material properties, high energy costs, and reduced economic benefits [17]. Thus, there is an urgent need to develop approaches to PET waste valorization in order to create valuable materials and address the problem of pollution [18]. Carbonization is a promising route for converting PET waste into valuable anode materials. During thermal treatment, the organic matrix of PET degrades to form a carbon phase [19] with relatively high yield and low ash content, as well as the possibility of fine-tuning the microstructure [20,21]. Carbonization parameters, primarily temperature and heating mode, determine the degree of graphitization, interlayer distance, pore distribution and volume, as well as the content and type of residual functional groups on the surface [22,23]. Therefore, the conversion of PET waste into HC anodes not only solves the plastic waste problem but also offers a sustainable, low-cost, and high-performance material for next-generation energy storage systems [24,25].

The aim of this work is to study the effect of the heat treatment temperature of recycled PET on the crystal structure, interlayer distance, presence of functional groups, and associated electrophysical properties of the resulting HC. Such research will help determine the optimal synthesis conditions for PET-HC for SIB anodes and substantiate the relationship between the structure of the material and its electrochemical characteristics.

## 2. Materials and Methods

### 2.1. Material Synthesis

**Figure 1:**
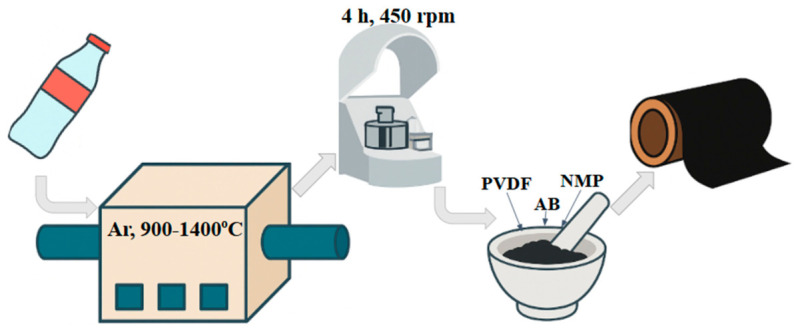
Schematic illustration of the synthesis process of hard carbon (HC) anode materials derived from waste PET bottles.

PET bottle waste was used as the raw material. The bottles were crushed into small pieces, and then thoroughly washed with water and ethanol to remove contaminants. The cleaned material was dried in an oven overnight. The dried fragments were subjected to heat treatment (carbonization) in a tube furnace at temperatures of 900 °C, 1000 °C, 1100 °C, 1200 °C, 1300 °C, and 1400 °C in an argon atmosphere. The heating rate was 2 °C/min, and the holding time at the set temperature was 1 h. The resulting carbon materials were designated according to the carbonization temperature: PET_900, PET_1000, PET_1100, PET_1200, PET_1300, and PET_1400. A schematic illustration of the synthesis procedure is shown in Figure 1.

### 2.2. Materials Characterization

The surface morphology of carbon samples was studied using scanning electron microscopy (SEM, JSM IT-800, JEOL Ltd., Tokyo, Japan). The microstructure of the material was further studied using transmission electron microscopy (TEM, JEM-ARM200F, JEOL Ltd., Tokyo, Japan). The crystal structure and interlayer distance were analyzed by X-ray diffraction (XRD, Rigaku Corporation, Tokyo, Japan). Raman spectroscopy was performed on a spectrometer (Horiba LabRam Evolution, Horiba Scientific, Kyoto, Japan) with a laser wavelength of 532 nm. The chemical composition of the surface and the valence states of the elements were determined by X-ray photoelectron spectroscopy (XPS, NEXSA, Thermo Fisher Scientific, East Grinstead, UK).

### 2.3. Electrochemical Characterization

The active material was mixed with polyvinylidene fluoride (PVDF) and acetylene black (AB) in a mass ratio of 8:1:1, with the addition of N-methyl-2-pyrrolidone (NMP) as a solvent, until a homogeneous slurry was obtained. The resulting suspension was evenly applied to copper foil and dried in a vacuum oven at 80 °C overnight. The electrodes had mass loadings of around 3–4 mg/cm^2^.

A Na//C half-cell was assembled for electrochemical testing in an inert atmosphere glove box (H_2_O < 0.5 ppm, O_2_ < 0.5 ppm). An Absorbed Glass Mat (AGM) was used as the separator, and a 1 M solution of NaPF_6_ in a mixture of ethylene carbonate (EC), dimethyl carbonate (DMC), and fluorethylene carbonate (FEC) solvents (volume ratio 49:49:2) was used as the electrolyte.

Galvanostatic cycling was performed in the voltage range of 0.01–2.5 V using a Neware test system (Neware Technology Limited, Shenzhen, China) at room temperature. Cyclic voltammetry (CV) measurements were carried out on a Biologic electrochemical workstation. Rate performance tests were conducted at various C-rates using a WonATech battery testing system (WonATech Co., Ltd., Seoul, Republic of Korea).

### 2.4. Use of Generative AI for Graphical Abstract Preparation

The graphical abstract was generated using ChatGPT (OpenAI, GPT-5.3) with image generation capabilities. The visual concept was developed based on the authors’ original scientific idea and translated into structured prompts describing the relationship between recycled plastic waste and anode material formation. An iterative prompt-refinement process was employed to achieve the desired scientific representation and visual clarity. No third-party images or copyrighted materials were used. The final graphical abstract was critically reviewed and edited by the authors to ensure scientific accuracy and consistency with the study.

## 3. Results

### 3.1. Material Characterization

**Figure 2:**
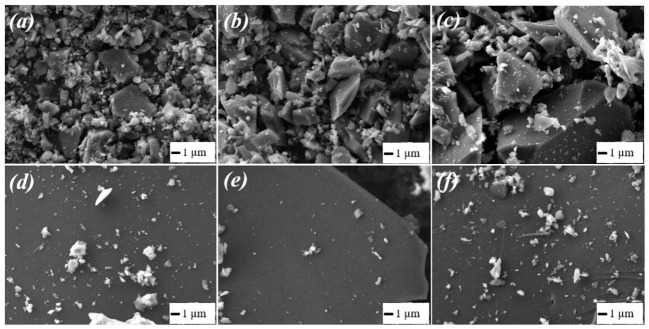
SEM images of PET-derived carbon samples: (**a**) PET_900, (**b**) PET_1000, (**c**) PET_1100, (**d**) PET_1200, (**e**) PET_1300, and (**f**) PET_1400.

SEM images at lower carbonization temperatures reveal no significant morphological changes (Figure 2a–c). Clusters of small particles exhibit an irregular morphology resembling microscopic rock-like fragments, which is characteristic of samples carbonized at 900–1100 °C. When the temperature is increased to 1200–1400 °C, SEM images (Figure 2d–f) show partial smoothing of the surface and the appearance of more flattened structural elements. In this temperature range, particle sintering and a reduction in interparticle voids become noticeable, indicating microstructure compaction [26]. As is common for HC derived from polymer precursors, the material generally stays partially porous and granular [27].

**Figure 3:**
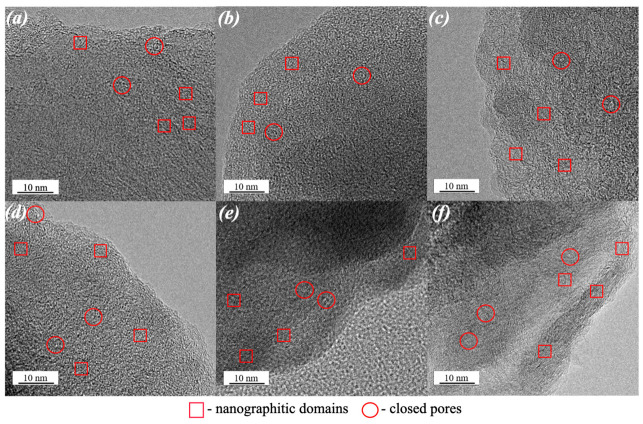
HRTEM images of PET-derived carbon samples: (**a**) PET_900, (**b**) PET_1000, (**c**) PET_1100, (**d**) PET_1200, (**e**) PET_1300, and (**f**) PET_1400.

Figure 3a–f displays HR-TEM images of the PET_900–PET_1400 samples. The microstructure of HC is characterized by a predominantly disordered carbon matrix with local nanographite domains and partially present closed pores. For PET_900 (Figure 3a), the initial formation of nanographite domains and separate closed pores is noted. In the PET_1000–PET_1100 (Figure 3b,c) samples, the nanographite domains are more widely and distinctly distributed, while partially closed pores are retained. In the 1200–1400 °C range (Figure 3d–f), the nanographitic domains are more developed and distinctly distributed.

**Figure 4:**
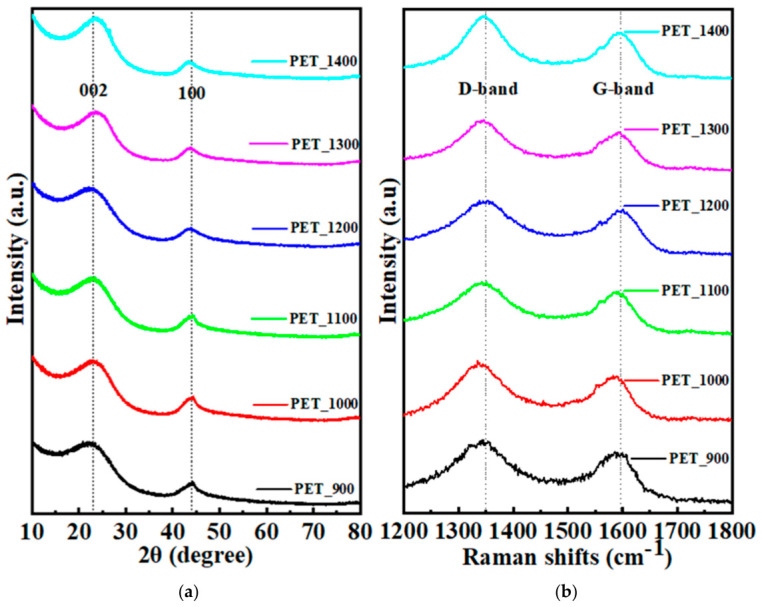
Structural characterization of PET-derived carbon samples: (**a**) XRD patterns and (**b**) Raman spectra.

Analysis of the XRD patterns confirms that all samples are hard carbon materials with an amorphous-turbostratic structure, as evidenced by two broad reflections at 2θ ≈ 22–25° and ≈ 43–44°, corresponding to the (002) and (100) planes of graphitic carbon [28]. The (002) peak of the carbon structure exhibits a complex evolution with increasing temperature, according to a comparative investigation of the XRD patterns of the PET_900–PET_1400 samples (Figure 4a). At 900 °C, the (002) maximum is located at 2θ ≈ 21.7° (d_002_ ≈ 0.410 nm).

Between 1000 and 1200 °C, the position of the (002) reflection remains essentially unchanged (Δ2θ < 0.5°), while d_002_ remains in the range of 0.387–0.393 nm. Despite the absence of statistically significant differences in d_002_ within the 1000–1200 °C range, the combination of relatively enlarged interlayer spacing and partial structural ordering in these samples may provide a favorable balance between Na^+^ diffusion and electronic conductivity [29]. A clear shift of the (002) reflection toward higher angles becomes evident only at 1300–1400 °C, where d_002_ decreases to approximately 0.377–0.381 nm, indicating structural densification and partial graphitic ordering. Two characteristic peaks are observed in Raman spectra (Figure 4b): the G band (~1580–1590 cm^−1^), which is brought on by the stretching of sp^2^ carbon bonds in graphite layers, and the D band (~1340–1350 cm^−1^), which is linked to defects and structural disruptions [25]. The I_D_/I_G_ ratio exhibits a nonlinear dependence on carbonization temperature. At 900 °C, 1100 °C, and 1200 °C, the ratio remains relatively stable at approximately 1.13, 1.14, and 1.15, respectively, indicating similar degrees of structural disorder. However, at 1000 °C the I_D_/I_G_ ratio markedly increases to ~1.29, suggesting enhanced defect density and edge site formation at this temperature. At higher temperatures of 1300 °C and 1400 °C, the ratio remains elevated (≈1.25 and 1.27), reflecting sustained structural disorder relative to the lower-temperature samples.

**Figure 5:**
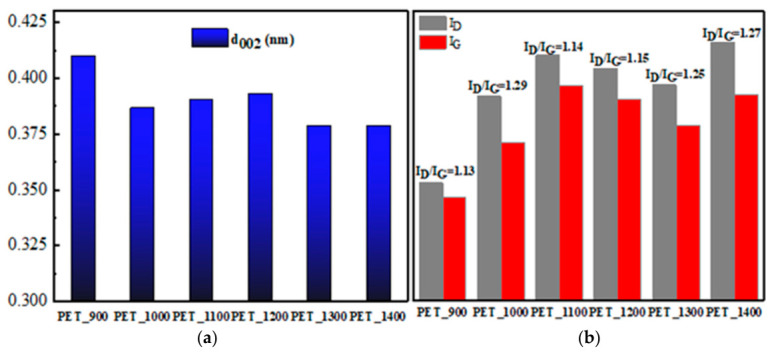
Structural parameters of PET-derived carbons: (**a**) d_002_ values; (**b**) I_D_, I_G,_ and I_D_/I_G_ ratio.

The structural parameters obtained from XRD and Raman analyses are summarized in Figure 5a,b. PET_900 exhibits the largest interlayer spacing (d_002_ ≈ 0.410 nm), characteristic of highly disordered hard carbon. Between 1000 and 1200 °C, d_002_ remains within a relatively narrow range (0.387–0.393 nm), and the observed variations fall within the uncertainty associated with determining the position of broad amorphous (002) reflections. In contrast, the Raman I_D_/I_G_ ratio varies more noticeably across this temperature range, indicating changes in defect structure and local carbon ordering that are not fully reflected by XRD-derived interlayer spacing alone. At higher temperatures (1300–1400 °C), a clear decrease in d_002_ to approximately 0.378 nm is observed, indicating structural densification and partial graphitic ordering. Simultaneously, the relatively high I_D_/I_G_ values suggest the persistence of defective and turbostratic carbon domains. Overall, the combined XRD and Raman results suggest that the structural evolution of PET-derived hard carbons during carbonization involves gradual rearrangement of defective carbon domains together with progressive ordering of carbon layers [30,31,32].

**Figure 6:**
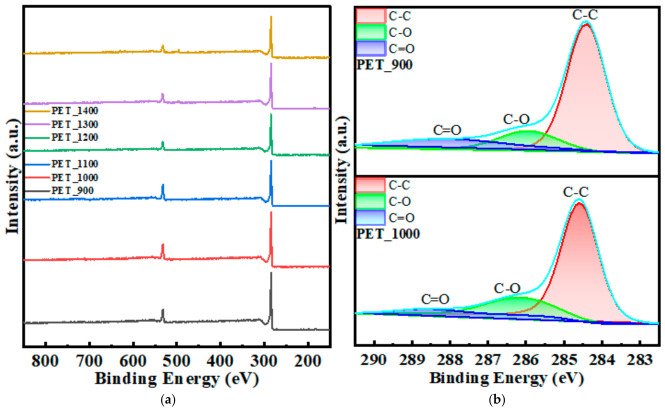
(**a**) XPS survey spectra of PET-derived carbons (PET_900–PET_1400); (**b**) deconvoluted C1s spectra of PET_900 and PET_1000.

Quantitative analysis of the survey XPS spectra (Figure 6a) revealed that the surface atomic percentage of carbon varies between 85.3 and 89.9 at. %, whereas the surface oxygen content ranges from 10.1 to 14.7 at. %. The C and O contents for the PET_900 sample are 88.3 and 11.7 at. %, respectively. The carbon percentage decreases to 86.7 and 85.3 at. % when the temperature is increased to 1000 and 1100 °C, respectively, while the oxygen fraction rises to 13.3 and 14.7 at.%. This behavior can be attributed to the partial preservation of oxygen-containing functional groups at intermediate carbonization temperatures. When the temperature is further increased to 1200–1400 °C, the carbon fraction increases to 89.4–89.9 at. % and the oxygen content decreases to 10.6–10.1 at. %, indicating more efficient elimination of oxygen functionalities and a higher degree of carbonization.

Figure 6b shows the deconvoluted C1s XPS spectra for the PET_900 and PET_1000 samples, demonstrating the contribution of carbon and oxygen-containing states. Deconvolution of the C1s spectrum reveals three components centered at ~284.4 eV (C-C), ~286 eV (C–O), and ~288.7 eV (C=O) [31], corresponding to different functional groups on the surface of the carbon material. Increasing the carbonization temperature from 900 °C to 1000–1100 °C leads to an increase in the proportion of C–C(sp^2^), C–O, and C=O bonds, which is reflected in an increase in the total oxygen content from ~11.7 at. % to ~13.3 and ~14.7 at.% for the samples, respectively. The corresponding C1s XPS spectra for PET_1100, PET_1200, PET_1300, and PET_1400 are shown in Figure S1. Above 1100 °C, a progressive attenuation of the C–O and C=O components is observed relative to the dominant C–C(sp^2^) peak, indicating thermal elimination of oxygen-containing surface groups at higher carbonization temperatures. Higher oxygen content and oxygen-containing functional groups on carbon anodes provide additional active sites for ion adsorption and improve wettability with the electrolyte, thereby enhancing the ion storage and electrochemical performance of the electrode [33,34].

### 3.2. Electrochemical Performance

**Figure 7:**
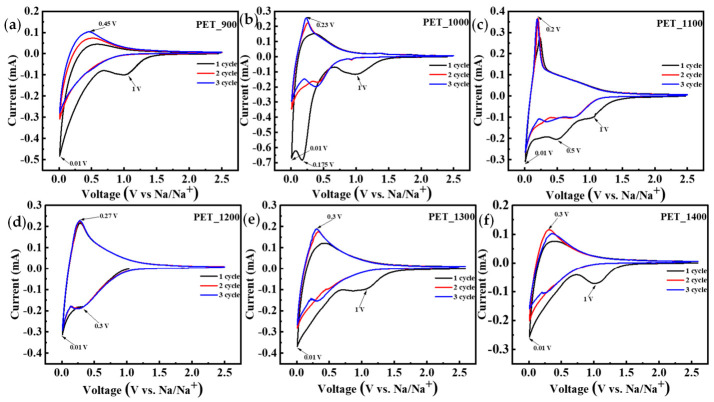
CV curves of PET-derived HC anodes: (**a**) PET_900, (**b**) PET_1000, (**c**) PET_1100, (**d**) PET_1200, (**e**) PET_1300, and (**f**) PET_1400.

Cyclic voltammetry of carbon samples PET_900–PET_1400 was performed in the range of 0–2.5 V relative to Na/Na^+^ for the first three cycles (Figure 7). For all samples, a pronounced broad peak in the range of 0.01–1 V was observed in the first cycle, associated with the decomposition of the electrolyte and the formation of the SEI film, as well as with the irreversible capture of Na^+^ by defective areas of the carbon matrix [35]. In subsequent cycles, the intensity of this peak decreases significantly, and the curves of the 2nd and 3rd cycles nearly overlap, which indicates stabilization of the electrode/electrolyte interface and good reversibility of the sodium intercalation/deintercalation processes [36]. PET_900 (Figure 7a) shows a broad anodic peak around ~0.45 V, suggesting that sodium storage is mainly governed by adsorption at defects and disordered regions [37]. In contrast, PET_1000 (Figure 7b) and PET_1100 (Figure 7c) display sharper and more intense peaks (~0.20–0.23 V), indicating more active and reversible Na^+^ storage [38]. With further increase in carbonization temperature 1200–1400 °C (Figure 7d–f), the anodic peak gradually shifts to ~0.27–0.30 V and becomes less intense, reflecting increased structural ordering and a reduced number of active sites [39]. Overall, the gradual shift of the anodic peak toward higher potential with increasing carbonization temperature suggests a transition from defect-dominated sodium adsorption to more ordered Na^+^ storage in graphitic domains [40].

**Figure 8:**
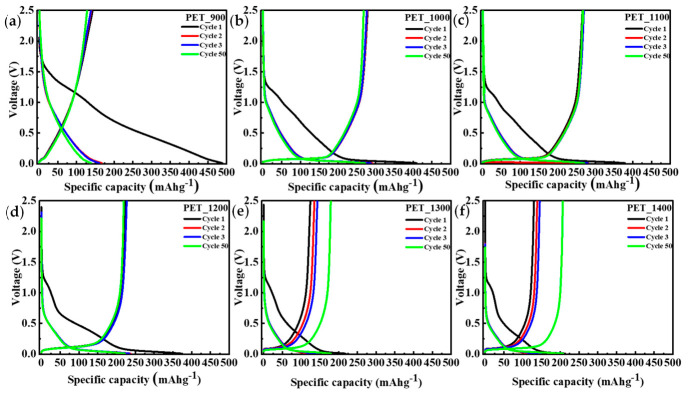
Galvanostatic charge–discharge profiles of PET-derived HC anodes: (**a**) PET_900, (**b**) PET_1000, (**c**) PET_1100, (**d**) PET_1200, (**e**) PET_1300, and (**f**) PET_1400 at a current density of 20 mA g^−1^.

The galvanostatic charge–discharge curves of anode materials obtained from PET at carbonization temperatures of 900–1400 °C are shown in Figure 8. Measurements were performed in the voltage range of 0.01–2.5 V relative to Na^+^/Na for the 1st, 2nd, 3rd, and 50th cycles. In the first cycle, significant irreversible capacity losses are observed, associated with the formation of the SEI film, electrolyte decomposition, and irreversible capture of Na^+^ ions in defective areas of the carbon matrix [41]. The discharge capacity of the first cycle decreases sequentially with increasing carbonization temperature and is approximately 500, 410–420, 380–385, 380, 225, and 210 mAh g^−1^ for the PET_900, PET_1000, PET_1100, PET_1200, PET_1300, and PET_1400 samples, respectively. The shape of the GCD profiles reveals a systematic evolution of the dominant Na^+^ storage mechanism with increasing carbonization temperature, reflecting contributions from surface/defect adsorption (sloping region, >0.1 V vs. Na^+^/Na), possible pseudocapacitive storage associated with oxygen-containing functional groups, and pore-related Na^+^ storage in the low-voltage plateau region (<0.1 V) [42,43,44]. PET_900 (Figure 8a) exhibits a dominant sloping region (1.3–0.2 V) associated with Na^+^ adsorption at defect sites and an almost negligible low-voltage plateau, indicating a small proportion of closed pores [42]. PET_1000 and PET_1100 (Figure 8b,c) display a pronounced plateau at ≈0.02 V due to the closed-pore filling and Na-cluster formation [43], whereas PET_1200 (Figure 8d) shows similar but less prominent behavior. In PET_1000 and PET_1100, the plateau region contributes approximately 40–50% of the total stable capacity, while the remaining capacity originates from the sloping region. This balance between multiple coexisting Na-storage contributions explains why PET_1000 and PET_1100 outperform higher-temperature samples despite PET_1000 exhibiting a higher ID/IG ratio (1.29) than PET_1200 (1.15). The elevated disorder at 1000 °C likely reflects the presence of active edge defects and turbostratic domains that can contribute to reversible Na^+^ adsorption rather than irreversible trapping. Concurrently, XPS analysis reveals elevated oxygen contents at 1000–1100 °C (13.3–14.7 at.%), suggesting the presence of oxygen-containing surface groups commonly associated with additional pseudocapacitive Na^+^ storage [33]. These structural features may contribute to the improved rate retention of PET_1000 and PET_1100 together with their relatively high ICE values (69–71%) and stable Coulombic efficiency exceeding 98% after the initial activation cycles. At 1200 °C, the plateau contribution becomes less pronounced concurrently with a reduction in oxygen content (10.6 at.%), suggesting that both pseudocapacitive and pore-related Na-storage contributions are reduced relative to 1000–1100 °C. The stabilization and near overlap of the curves from the 2nd to the 50th cycle indicate the formation of a stable SEI layer and the high reversibility of the sodium insertion/deinsertion processes [44]. In PET_1300 (Figure 8e) and PET_1400 (Figure 8f), the low-voltage plateau is significantly weakened due to the development of more ordered nanographitic domains and progressive structural densification. The plateau contribution decreases with increasing carbonization temperature, indicating that pore-related Na^+^ storage becomes less significant. At higher temperatures, interlayer Na^+^ insertion into more ordered carbon domains may play a relatively larger role. However, this does not compensate for the overall decrease in reversible capacity, which suggests a reduction in accessible Na-storage sites. Overall, the best electrochemical performance of PET-derived hard carbons is obtained at intermediate carbonization temperatures, where different Na-storage mechanisms coexist in a partially ordered turbostratic structure [45].

**Figure 9:**
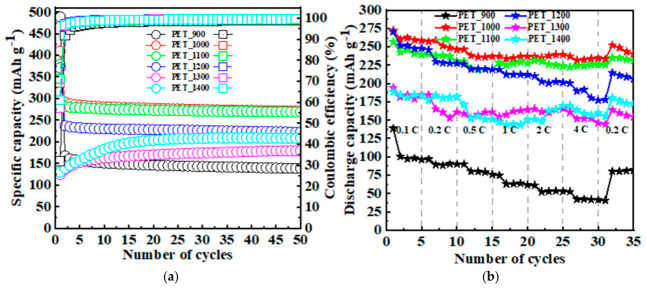
(**a**) Cycling stability and (**b**) rate capability of PET-derived carbon anodes carbonized at 900–1400 °C.

The cycling performance of HC anodes made from PET at 20 mA g^−1^ over 50 cycles is displayed in Figure 9a. After the initial irreversible losses associated with SEI formation, the samples stabilize to different reversible capacities [46]: PET_1000 and PET_1100 exhibit the best performance with stable capacities of ≈280 and ≈275 mAh g^−1^, respectively, together with the highest ICE values of 69.2% and 71.0%. PET_1200, PET_1400, and PET_1300 show lower stabilized capacities of ≈235, ≈180, and ≈200 mAh g^−1^, while PET_900 remains the lowest at ≈135 mAh g^−1^. All samples display a pronounced first-cycle capacity loss followed by a rapid increase in Coulombic efficiency, which reaches ≈96–100% within the first few cycles. The decrease in capacity for samples carbonized at higher temperatures is attributed to the loss of active sites and microstructural changes [47], whereas the superior behavior of PET_1000 and PET_1100 reflects an optimal balance between disorder and accessible storage sites.

The rate capability of PET_900–PET_1400 electrodes relative to Na/Na^+^ at different current densities is shown in Figure 9b. Good electrochemical reversibility is demonstrated by the discharge capacity, which falls with increasing current density and partially recovers when the rate is restored to 0.2 C. With the largest reversible capacities and greater rate tolerance, PET_1000 and PET_1100 exhibit the best overall performance of all the samples. At 0.1 C, PET_1000 shows an initial discharge capacity of around 265 mAh g^−1^. This capacity somewhat decreases to ~240–250 mAh g^−1^ at higher rates, and recovers to ~253 mAh g^−1^ when the current density is returned to 0.2 C. Similarly, PET_1100 has outstanding rate capability and reversibility, displaying ~240 mAh g^−1^ at 0.1 C, maintaining stable capacities around ~225 mAh g^−1^ throughout a wide range of current densities (1–5 C), and recovering to ~235 mAh g^−1^ at the last 0.2 C step. PET_1200, on the other hand, shows a somewhat reduced capacity (from ~250 mAh g^−1^ at 0.1 C to ~225 mAh g^−1^ at 0.2 C), a more noticeable fall at higher rates, and a partial recovery in the last low-rate phase. Despite exhibiting reversible behavior when the current density is lowered, PET_1300 and PET_1400 suffer from significantly diminished capacities (usually in the range of ~150–190 mAh g^−1^). The PET_900 sample shows the poorest performance, with the discharge capacity rapidly decreasing from ~137 mAh g^−1^ at 0.1 C to below 100 mAh g^−1^ at higher rates and remaining at ~88 mAh g^−1^ after recovery, indicating insufficient formation of electrochemically active carbon structure at this carbonization temperature.

The electrochemical performance of PET_1000 and PET_1100 was further contextualized against existing waste-derived hard carbon anodes reported in the literature (Table 1). While PET-based materials with ZnO template engineering [12] exhibit higher absolute capacities (389 mAh g^−1^), rate capability data for these systems are not reported, and their synthesis relies on additional chemical and structural engineering. In comparison, lower rate retention values are observed for waste towel-derived HC (68.9%) [47], pomegranate peel-derived HC (52.7%) [48], and N-doped corn stalk HC (47.9%) [49], all of which involve multi-step processing or chemical pre-treatment/doping strategies. These results indicate that simple, additive-free pyrolysis of waste PET at 1000–1100 °C can achieve competitive rate performance compared to more complex preparation routes reported in the literature. Among reported hard carbon anodes for which rate performance can be directly compared under similar conditions, PET_1000 and PET_1100 show the highest rate retention values (89.9% and 93.0% at 1000 mA g^−1^, respectively).

Beyond electrochemical performance, the use of waste PET as a carbon precursor offers tangible practical advantages. The raw material cost of post-consumer PET is effectively negligible, as it represents an end-of-life plastic waste stream requiring disposal, in contrast to synthetic precursors or commercial hard carbon products that represent a primary cost barrier to sodium-ion battery commercialization [50]. The carbon yield of PET under inert pyrolysis is competitive with typical biomass-derived carbons while avoiding the compositional variability inherent to natural precursors [20,21]. The one-step, additive-free carbonization employed in this work requires no chemical activation agents or structural templates, unlike more complex PET-derived HC routes involving ZnO mesopore engineering [12] or ionothermal synthesis [25], reducing process complexity and eliminating associated chemical waste streams. From an environmental standpoint, direct carbonization of PET waste simultaneously addresses two pressing challenges: diversion of post-consumer plastic from landfill and incineration, and supply of competitive anode materials for sodium-ion batteries targeting large-scale stationary energy storage [22]. Considering the large global production of PET [11], even partial valorization of this waste stream into battery-grade hard carbon represents a meaningful contribution to circular economy goals.

## 4. Conclusions

This paper proposes a sustainable approach to converting PET waste into HC anode material for SIBs through direct carbonization in the range of 900–1400 °C. It has been established that the carbonization temperature significantly affects the electrochemical properties of the material. The PET_1000 and PET_1100 samples demonstrated an optimal combination of characteristics, providing a stable reversible capacity of ~275–280 mAh g^−1^ at a current density of 20 mA g^−1^ and good cycle stability. The initial irreversible capacity loss is associated with the formation of the SEI layer and is typical for HC anodes. The results confirm the promise of recycled PET as an affordable and sustainable precursor for the creation of competitive anode materials for sodium-ion energy storage systems.

## Figures and Tables

**Table 1:** Comparison of the electrochemical performance of PET-derived hard carbon anodes obtained in this work with waste-derived hard carbon anodes from the literature.

Precursor	Reversible Capacity	ICE (%)	Rate Retention (%)	Ref.
Waste PET, 1000 °C	259 at 20 mA g^−1^	69.2	89.9	This work
Waste PET, 1100 °C	270 at 20 mA g^−1^	71	93	This work
PET + ZnO template	389 at 20 mA g^−1^	-	-	[12]
Waste towel, 1300 °C	341 at 64 mA g^−1^	75.4	68.9	[47]
Pomegranate peel, 1100 °C	330 at 33 mA g^−1^	-	52.7	[48]
N-doped corn stalk	259 at 100 mA g^−1^	65.7	47.9	[50]

## Data Availability

The original contributions presented in this study are included in the article/Supplementary Material. Further inquiries can be directed to the corresponding authors.

## Supplementary Materials

The following supporting information can be downloaded at: https://www.mdpi.com/article/10.3390/ma19122457/s1. Figure S1: Deconvoluted C1s spectrum of (a) PET_1100, (b) PET_1200, (c) PET_1300, (d) PET_1400.

## Abbreviations

The following abbreviations are used in this manuscript:

SIB	Sodium-ion batteries
LIB	Lithium-ion batteries
PET	Polyethylene terephthalate
HC	Hard carbon
SEM	Scanning electron microscopy
TEM	Transmission electron microscopy
XRD	X-ray diffraction
XPS	X-ray photoelectron spectroscopy
PVDF	Polyvinylidene fluoride
AB	Acetylene black
NMP	N-methyl-2-pyrrolidone
AGM	Absorbed Glass Mat
EC	Ethylene carbonate
DMC	Dimethyl carbonate
FEC	Fluorethylene carbonate
CV	Cyclic voltammetry
