# Supplementary material for: PET Waste-Derived Hard Carbon with Superior Rate Capability for Sodium-Ion Battery Anodes
Source: Materials (Basel). 2026 Jun 8;19(12):2457. doi: 10.3390/ma19122457 (PMC13302349; doi:10.3390/ma19122457)
Supplement: Supplementary file 1 [file materials-19-02457-s001.zip › materials-4288576-supplementary.pdf]

Supplementary File

PET Waste–Derived Hard Carbon with Superior Rate Capability for Sodium-Ion Battery Anodes

Aizhuz Sarsengaliyeva[1], Aliya Mukanova[1,2], Sung-Soo Kim[3*], Arailym Nurpeissova[1,2*]

[1]Institute of Batteries (IoB), Kabanbay batyr ave. 53, Astana, 010000, Kazakhstan
[2]Institute of New Materials and Energy Technologies, Kabanbay batyr ave. 53, Astana, 010000, Kazakhstan
[3]Graduate School of Energy Science and Technology, Chungnam National University, 99 Daehak-ro, Yuseong-gu, Daejeon 34134, South Korea

*Correspondence: arailym.nurpeissova@nu.edu.kz

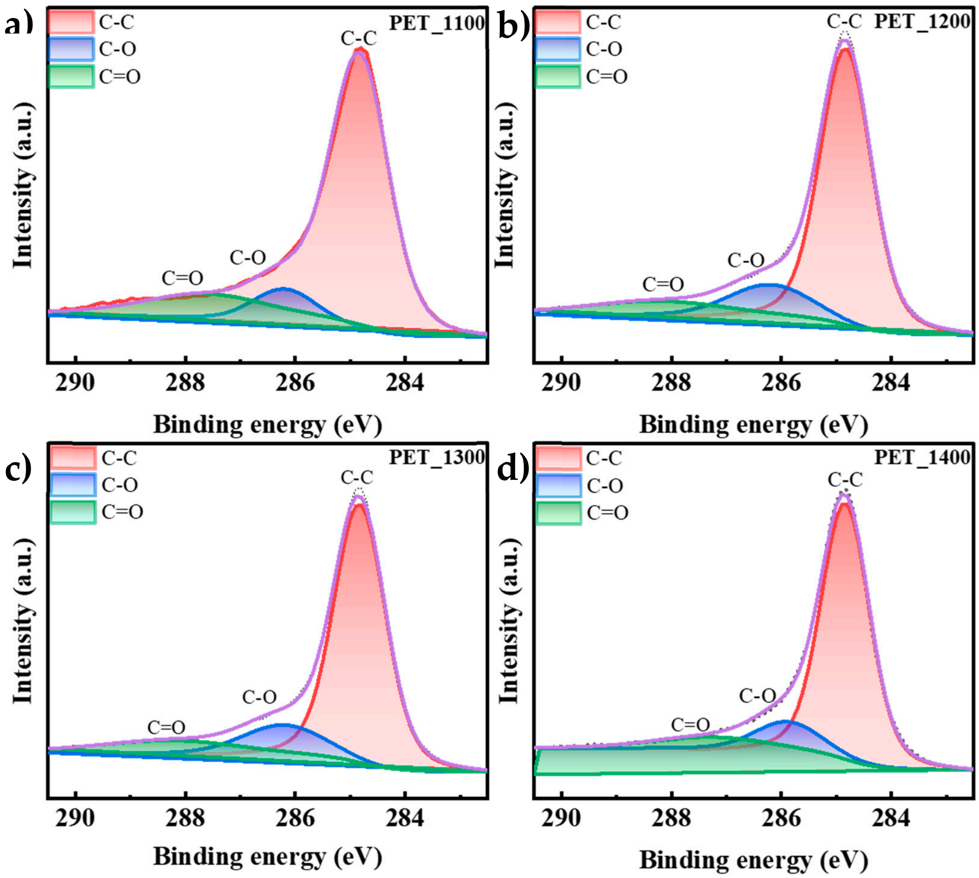

Figure S1. Deconvoluted C1s spectrum of (a) PET_1100, (b) PET_1200, (c) PET_1300, (d) PET_1400
